# Effect of β-alanine treatment on mitochondrial taurine level and 5-taurinomethyluridine content

**DOI:** 10.1186/1423-0127-17-S1-S25

**Published:** 2010-08-24

**Authors:** Chian Ju Jong, Takashi Ito, Mahmood Mozaffari, Junichi Azuma, Stephen Schaffer

**Affiliations:** 1 Department of Pharmacology, University of South Alabama, College of Medicine, Mobile, Alabama 36688, USA; 2 Clinical Evaluation of Medicines and Therapeutics, Graduate School of Pharmaceutical Sciences, Osaka University, Osaka 565-0871, Japan; 3 Department of Oral Biology, Medical College of Georgia, School of Dentistry, Augusta, Georgia, USA

## Abstract

**Background:**

The β-amino acid, taurine, is a nutritional requirement in some species. In these species, the depletion of intracellular stores of taurine leads to the development of severe organ dysfunction. The basis underlying these defects is poorly understood, although there is some suggestion that oxidative stress may contribute to the abnormalities. Recent studies indicate that taurine is required for normal mitochondrial protein synthesis and normal electron transport chain activity; it is known that defects in these events can lead to severe mitochondrial oxidative stress. The present study examines the effect of taurine deficiency on the first step of mitochondrial protein synthesis regulation by taurine, namely, the formation of taurinomethyluridine containing tRNA.

**Methods:**

Isolated rat cardiomyocytes were rendered taurine deficient by incubation with medium containing the taurine transport inhibitor, β-alanine. The time course of cellular and mitochondrial taurine depletion was measured. The primer extension method was employed to evaluate the effect of β-alanine treatment on taurinomethyluridine content of tRNA^Leu^. The protein levels of ND6 were also determined by Western blot analysis.

**Results:**

β-alanine caused a time-dependent decrease in cellular taurine content, which were reduced in half after 48 hrs of incubation. The amount of taurine in the mitochondria was considerably less than that in the cytosol and was unaffected by β-alanine treatment. Approximately 70% of the tRNA^Leu^ in the untreated cell lacked taurinomethyluridine and these levels were unchanged following β-alanine treatment. Protein content of ND6, however, was significantly reduced after 48 hours incubation with β-alanine.

**Conclusions:**

The taurine levels of the cytosol and the mitochondria are not directly coupled. The β-alanine-mediated reduction in taurine levels is too small to affect taurinomethyluridine levels. Nonetheless, it interferes with mitochondrial protein synthesis, as exemplified by a decrease in ND6 protein content. Thus, β-alanine does not cause alterations in mitochondrial protein synthesis through the lowering of taurine levels.

## Background

Taurine is a β-amino acid found in high concentrations in most tissues, with levels being particularly high in excitable tissue [1-3]. Although taurine was identified as a component of biological material as early as 1827, its physiological functions have not been adequately identified. Among the recognized actions of taurine are osmoregulation, membrane modulation, antioxidation, cytoprotection, anti-inflammation and regulation of ion transport [4-8]. Many of these actions are attributed to one or more of its biochemical reactions, which include conjugation [4], modulation of protein phosphorylation status [5], alteration in phospholipid N-methylation [6], neutralization of hypochlorous acid [9] and regulation of ion transport [10].

The link between the biochemical reactions of taurine and the overt consequences of taurine deficiency, such as immune deficiency, stunted growth, birth defects and the development of a retinopathy and cardiomyopathy [7,11-14], remain unclear. Because many cardiomyopathies are initiated by oxidative damage, the suggestion that excessive reactive oxygen species (ROS) production may contribute to the development of the taurine deficient cardiomyopathy seems reasonable [8]. However, the mechanism by which taurine modulates oxidative stress has been enigmatic. Not only is taurine incapable of functioning as a classical free radical scavenger [15], but there is no concrete evidence that it upregulates the antioxidant defenses of the cell. Recently, it has been proposed that taurine might indirectly modulate the production of ROS by the mitochondria [16]. According to the proposal of Schaffer *et al.*[16], the initial step in taurine deficiency-mediated ROS generation is a decline in the 5-taurinomethyluridine content of mitochondrial tRNA^Leu^. Because the conjugation of taurine with a key uridine moiety of mitochondrial tRNA^Leu^ enhances the binding of tRNA to the codon of mRNA [17], decreases in 5-taurinomethyluridine content lead to diminished rates of mitochondrial encoded protein synthesis. According to Kirino *et al.*[17], translational deficiencies develop in tRNA^Leu^ containing the anticodon, UAA, and lacking 5-taurinomethyluridine. Because the UAA anticodon interacts weakly with the codon UUG but not to the codon UUA, the translational defect only occurs with mRNAs containing the UUG codon. In turn, lower rates of protein synthesis interfere with the normal assembly of the respiratory chain complexes, which consist of both nuclear and mitochondria encoded proteins. It is well recognized that reduced levels of the respiratory chain complexes result in impaired flux of electrons through the electron transport chain. Consequently, the efficiency of the electron transport chain declines, as electrons are diverted from the electron transport chain to oxygen, forming in the process superoxide. There is some precedence for this hypothesis. Kirino *et al.*[18] observed that a group of patients with symptoms of mitochondrial myopathy, encephalopathy, lactic acidosis and stroke-like episodes (MELAS) contain several mitochondrial tRNA mutations, which lead to a lack of the 5-taurinomethyluridine moiety.

A major assumption of the Schaffer hypothesis [16] is that a decline in intracellular taurine levels inevitably leads to a decrease in the formation of 5-taurinomethyluridine. However, very little information is available on the relationship between cellular taurine levels and mitochondrial taurine content. Moreover, the dependence of 5-taurinomethyluridine formation on mitochondrial taurine levels remains to be established. Further, the assumption that reduced 5-taurinomethyluridine levels decrease the expression of a protein, whose mRNA contains high levels of the UUG codon, needs further study. To evaluate the validity of these assumptions, the present study examines the effect of drug-mediated taurine depletion on mitochondrial taurine content and the conjugation of mitochondrial tRNAs in isolated neonatal cardiomyocytes. Also examined is the expression of ND6, whose mRNA contains 8 UUG codons [17]. To decrease cellular taurine content, isolated cardiac cells were exposed to the taurine transport inhibitor, β-alanine [19-21]. Previous studies have shown that β-alanine-mediated taurine deficiency is associated with impaired rates of cardiomyocyte relaxation, changes in cell size and alterations in calcium movement [21,22].

## Methods

### Cardiomyocytes preparation and incubation conditions

Neonatal cardiomyocytes were prepared from 2-3-day old Wistar rats as described previously by Grishko *et al*. [23]. The use of animals for this study was approved by the Institutional Animal Care and Use Committee. Hearts were removed and the ventricular tissue was minced. Cells were isolated by four 30-minute cycles of enzymatic digestion with a mixture of trypsin, chymotrypsin and elastase (Calbiochem) in HEPES-buffered saline solution at 37^o^C. After dissociation, cells were pre-plated on plastic culture dishes for 90 minutes at 37^o^C to allow for nonmyocyte attachment. After the incubation period, the number of cells in the myocyte suspension was counted with a hemacytometer. The unattached cells were then suspended in minimum essential medium (MEM), containing 10% newborn calf serum and 0.1 mM 5-bromo-2-deoxyuridine. The cells were plated and incubated overnight to allow for attachment of viable cardiomyocytes. The serum-containing medium was replaced the next day with serum-substitute. The neonatal cardiomyocyte culture was maintained for 2 days at 37^o^C prior to being subjected to medium containing 0 mM (Control) for 48 hours or 5 mM β-alanine for 4 hours, 16 hours, 24 hours or 48 hours.

### Measurement of intracellular taurine levels

In order to measure intracellular taurine content, cells were first scraped from the dish and homogenized in ice cold 1 M perchloric acid and 2 mM EDTA. Homogenates were centrifuged at 10,000 g for 10 minutes at 4 ^0^C. The supernatant was neutralized to pH 5-6 using 2M KOH and centrifuged again at 10,000 g for 10 minutes at 4 ^0^C. The resulting supernatant (2-5 ml) was passed through Dowex-50 X 8 (200-400 mesh, 9 X 40-55 mm) columns and eluted with 2 ml of deionized water. An aliquot of the eluant was used to measure taurine levels using the procedures described by Shaffer and Kocsis [24]. A taurine standard curve was developed using 2-40 µg taurine for each assay.

### Primer extension method

The primer extension method described by Kirino *et al*. (18) was used to detect changes in the levels of 5-taurinomethyluridine of mitochondrial tRNA^Leu^ in β-alanine-treated cells. Total RNA was first isolated from cells using Trizol LS Reagent (Invitrogen; Carlsbad, CA) according to the protocol. The RNA samples were incubated at 80 ^0^C for 2 minutes in 10 mM Tris-HCl pH 8.0 buffer containing 1 mM EDTA and a ^32^P-labeled primer of mitochondrial tRNA^Leu^ (0.1 pmol of 5’-ACCTCTGGGAAGG-3’). The samples were then allowed to cool at room temperature for 1 hour. To initiate the primer extension reaction, RNase-free H_2_O, 5X reaction buffer required for reverse transcription reactions, a d/ddNTP mixture containing dATP, dTTP and ddGTP, 25 mM MgCl_2_ and Moloney murine leukemia virus reverse transcriptase were added. The mixture was incubated at 42 ^0^C for 1 hour. The reaction mixture was subjected to polyacrylamide gel electrophoresis containing 7 M urea. The radiolabeled bands were visualized by autoradiograph.

### Western blot analysis

To detect a specific protein, western blot analysis was performed according to the protocol described by Grishko *et al*. [23]. Cells were washed twice in buffer containing 250 mmol/L sucrose, 10 mmol/L triethanolamine, pH 7.6, at room temperature. Next, cells were lysed in ice cold isolation buffer. The homogenate was centrifuged at 800 g for 6 minutes at 4^0^C and the supernatant was retrieved and centrifuged again at 16,000 g for 20 minutes at 4^0^C. The pellet was defined as the mitochondrial fraction and used to determine ND6 and succinate dehydrogenase content. The pellet was suspended in RadioImmuno Precipitation Assay (RIPA) lysis buffer. Protein concentration was determined by the bicinchoninic acid (BCA) method. The mitochondrial sample was mixed with an equal volume of 5X electrophoresis sample buffer and the samples were then boiled for 3 minutes before being analyzed. During Western blot analysis, mitochondrial samples were subjected to one-dimensional electrophoresis using 12% SDS PAGE. The proteins were then transferred to nitrocellulose membranes and the membranes were blocked. After incubation with the appropriate antibody, the membranes were washed and then incubated with a secondary antibody. The Western blots were analyzed by the enhanced chemiluminescence reaction.

## Results

### Effect of β-alanine on intracellular taurine levels

**Figure 1:**
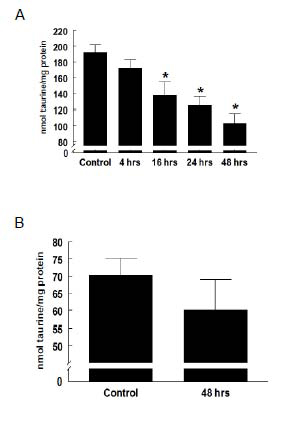
**Effect of β-alanine treatment on taurine content** Isolated neonatal cardiomyocytes were incubated with 5 mM β-alanine for various period of time. Intracellular taurine levels were determined as described in the methods. (A) β-alanine treatment reduces intracellular taurine content. Values shown represent means ± S.E.M. of 4-5 preparations. *Significant difference between β-alanine-treated groups and Control (*p*<0.05). (B) Mitochondrial taurine levels were unaffected by β-alanine treatment. Values shown represent means ± S.E.M. of 5-6 preparations.

Exposure of isolated neonatal cardiomyocytes to taurine transport inhibitor, β-alanine (5mM) resulted in a time-dependent decline in intracellular taurine levels (Figure 1A). Following 4 hours of β-alanine treatment, cellular taurine content declined only 10%. The rate of taurine depletion subsequently accelerated, with levels reaching 50% of normal by 48 hours of treatment. When normalized relative to protein content, the mitochondrial content of taurine was ~30% of total cellular levels. Surprisingly, mitochondrial taurine level was unaffected by the exposure of the cells to β-alanine (Figure 1B).

### Effect of taurine depletion on taurine conjugation of mitochondrial tRNA^Leu^

**Figure 2:**
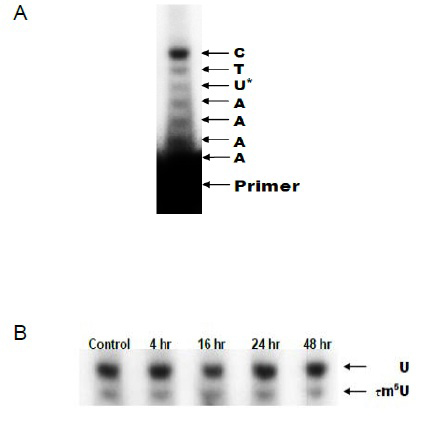
**Effect of β-alanine-mediated taurine depletion on wobble uridine modification** (A) The primer extension method was used to detect levels of 5-taurinomethyluridine in mitochondrial tRNA^Leu(UUR)^ before and following β-alanine-treatment. Shown is a representative gel of reverse transcription from the primer. A defect in uridine modification resulted in the extension of reverse transcription through the unmodified uridine (U*), which terminated at cytosine (C) due to the inclusion of dideoxyguanosine (ddGTP) in the reaction mixture. (B) Exposure of cardiomyocytes to β-alanine at various time intervals did not cause a significant change in either modified (τm^5^U) or unmodified uridine (U).

Using the primer extension method, we found that β-alanine-mediated taurine depletion did not affect the taurinomethyluridine content of mitochondrial tRNA^Leu(UUR)^ (Figure 2). Because modified uridine (τm^5^U) hinders the reverse transcription reaction, the primer extension method readily detects changes in 5-taurinomethyluridine content. While extension of the primer terminates one base after the modified uridine in the case of tRNA containing modified uridine, if the tRNA lacks the 5-taurinomethyluridine moiety, primary extension proceeds beyond the unmodified uridine (U). Figure 2A shows each species formed when the reverse transcriptase is added to medium containing a mixture of primer, tRNA^Leu(UUR)^, dTTP, dATP and ddGTP, the latter which terminates the reverse transcriptase reaction. Because the primer is complimentary to the TGGAGACCCTTCC sequence of the tRNA, the extension reaction adds TTTTAA to the primer of the τm^5^U form of tRNA but TTTTAAG to the U form of tRNA. As seen in Figure 2B, two primer extension species are produced, which show up on the gel as two specific bands. The U band represents primer from tRNA containing unmodified uridine and the τm^5^U band represents primer from tRNA containing modified uridine. In the untreated cells, approximately 1/3 of tRNA^Leu(UUR)^ contains modified uridine and the remainder unmodified uridine. Figure 2B also shows that β-alanine treatment had no effect on the relative levels of the U and τm^5^U forms of tRNA^Leu(UUR)^.

### Effect of taurine deficiency on ND6 protein level

**Figure 3:**
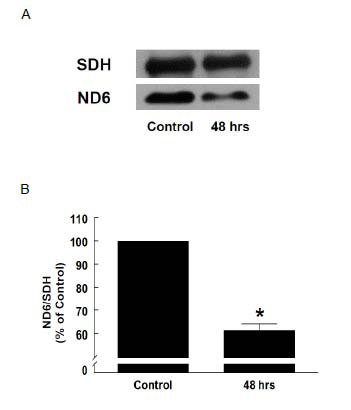
**Effect of taurine deficiency on protein levels of ND6** Cardiomyocytes were exposed to medium containing 5 mM β-alanine for 48 hours. Mitochondria were subjected to Western blot analysis of ND6. (A) Shown is a representative gel of ND6 and succinate dehydrogenase (SDH). (B) Values shown represent means ± S.E.M. of the ND6/SDH ratio of 6-7 preparations. *Significant difference between β-alanine-treated group and Control (*p*<0.05).

Our western blot analysis showed a reduction in the expression of ND6, a subunit of complex I of the respiratory chain, in β-alanine-treated cells. As shown in Figure 3, a significant reduction of 60% was observed at 48 hours, indicating that β-alanine decreases the expression of a mitochondrial-encoded protein, whose mRNA contains a high content of the UUG codon. A representative gel of ND6 is also shown in Figure 3.

## Discussion

The most important finding of this study is that drug-mediated taurine depletion initiates a time-dependent decline in cellular taurine content that is not associated with a decline in either mitochondrial taurine content or the 5-taurinomethyluridine content of mitochondrial tRNA^Leu^. Yet, we found that β-alanine treatment induces a significant reduction in mitochondrial protein expression of ND6. Based on the results of the primer extension procedure, we also conclude that ~67% of the tRNA^Leu^ in the untreated cells lacks the 5-taurinomethyluridine moiety. Based on the relative amount of primer that is not terminated at the taurinomethyluridine step, less than 33% of tRNA^Leu^ in the control cells contains 5-taurinomethyluridine (Figure 2). Moreover, exposure of the cells to medium containing the taurine transport inhibitor, β-alanine (5 mM), has no apparent effect on the taurinomethyluridine content of tRNA^Leu^.

The present study also shows that mitochondrial taurine content pales in comparison with taurine content of the whole cell. This is not surprising because taurine serves as an important osmolyte in the heart, which necessitates a high concentration of the β-amino acid in the cytosol [25]. However, Suzuki *et al.*[26] found that mitochondrial tRNA^Leu^ isolated from HeLa cells grown in medium containing ^18^O-taurine is labeled with ^18^O-taurinomethyluridine, indicating that 5-taurinomethyluridine levels are maintained in part by extracellular taurine. Moreover, taurine is readily taken up by isolated bovine mitochondria [26]. Nonetheless, the present study uncovered an uncoupling between changes in taurine content of the cytosol and the mitochondria. While whole cellular taurine levels declined in a time dependent manner by as much as 50% upon exposure to β-alanine, mitochondrial taurine content remained unaffected (Figure 1). This finding suggests that either the decline in cytosolic taurine has no influence on mitochondrial taurine uptake or the turnover of taurine by the mitochondria is very low. The former seems more reasonable, based on the work of Huxtable and Bressler [27], who found that very little ^14^C-taurine injected into rats are distributed to the mitochondria while large amounts end up in the cytosol.

The mechanism by which taurine enters the mitochondria from the cytoplasm is completely unknown. Although Suzuki *et al.*[26] first identified the existence of a mitochondrial transport event, they failed to characterize it. Nonetheless, it is logical to assume that the mitochondrial transporter is mechanistically and structurally different from the most widely studied taurine transporter, the Na^+^/Cl^-^ dependent transporter located on the cell membrane of a wide range of cell types [28-30]. A key difference between the two transporters lies in the need to transport taurine against a huge concentration gradient in the case of the cell membrane transporter vs. little if any gradient in the case of the mitochondrial transporter. Although taurine transport was not examined in the present study, we showed that the decrease in cytosolic taurine levels mediated by β-alanine treatment only reduce the concentration of cellular taurine from ~30 mM to ~15 mM. Because most transporters are characterized by Km values in the μM to low mM range, the mitochondrial transporter is likely to have a Km for taurine in the 1-10 mM range. The high cytosolic taurine levels may explain the failure of β-alanine treatment to affect mitochondrial taurine content, as 15 mM taurine would likely saturate the taurine binding sites of the mitochondrial transporter. However, it remains to be determined if more severe cytosolic taurine deficiency is capable of affecting mitochondrial taurine levels.

Kirino and colleagues [17] have suggested that the conjugation of mitochondrial tRNA by taurine ensures the proper expression of mitochondrial proteins. According to their theory, proteins whose mRNA contains large amounts of the UUG codon would be particularly sensitive to reduced taurine conjugation of tRNA^Leu^. The mRNA of ND6 contains 8 UUG codons. In fact, 42% of the leucine codons of ND6 are UUG codons. Therefore, the present study showing that β-alanine-treated cells exhibit a significant decline in ND6 protein levels (Figure 3) is in apparent agreement with the idea that taurine is essential for normal protein synthesis. However, β-alanine was not associated with a significant decrease in mitochondrial taurine and presumably no change in 5-taurinomethyluridine levels, which is in disagreement with the hypothesis of Kirino *et al.*[17]. These data imply that β-alanine acts at some other site in the synthesis of ND6. A logical possibility is that β-alanine conjugates with uridine, a reaction that could weaken the anticodon-codon interaction involving tRNA^Leu^. Therefore, β-alanine might interfere with the actions of taurine by weakening the U:G wobble base pairing, thereby reducing the synthesis of ND6.

A key question is whether the present findings provide insight into the development of pathological lesions in the taurine deficient cell. It has been shown that treatment of animals or cells with one of the taurine transport inhibitors, β-alanine or guanidinoethanesulfonate, decrease taurine levels by nearly 50% and lead to the development of specific pathological lesions. However, nutritional depletion of taurine in some species, such as fox, cats, and monkeys, result in the loss of up to 90-95% of myocardial taurine and lead to the development of overt cardiomyopathy [14,31] and retinopathy [12], severe developmental defects [13] and immunological deficiencies [7]. Fox, cats, some breeds of dogs and monkeys share two characteristics that drive severe taurine depletion and the appearance of cardiac and retinal lesions. First, they lack a robust pathway for taurine biosynthesis [32,33]. Second, they preferentially utilize taurine to conjugate bile acids [32,33]. Because bile acids serve as the most abundant sink for taurine and are lost on a daily basis in the feces, an imbalance readily develops between the supply and loss of taurine in the nutritionally deficient cat. Interestingly, humans lack a robust taurine biosynthetic pathway, but they can substitute glycine for taurine in the conjugation of bile acids; the bile acid requirement spares humans severe taurine deficiency and its pathological consequences [34].

The rat readily synthesizes taurine in the liver [35]. Because the heart lacks a robust taurine synthetic machinery, the heart depends upon taurine transport to maintain high intracellular levels. Huxtable [35] has shown that the taurine transport inhibitors diminish cardiac taurine levels by blocking both taurine transport and biosynthesis. One of these inhibitors, guanidinoethanesulfonate, has been shown to cause contractile dysfunction and the loss of myofibrils in the heart of treated rats [36,37]. However, guanidinoethanesulfonate diminishes the levels of creatine phosphate, an important energy metabolite in the heart, raising questions about the appropriateness of using guanidinoethanesulfonate to study the effects of taurine deficiency on heart function [38]. The other major taurine transport inhibitor, β-alanine, causes modest defects in myocardial relaxation, which have been attributed to impaired Ca^2+^ handling by the sarcoplasmic reticulum [22].

The damage caused by taurine deficiency in the nutritionally deficient cat is much more severe. Pion *et al.*[14] and Novotny *et al.*[32] have shown that cats fed a taurine deficient diet develop a severe cardiomyopathy, characterized by dilation of the left ventricle chamber and both systolic and diastolic dysfunction. Because myocardial taurine levels are decreased by >99% in these animals [32], it would be interesting to assess the effects of this severely depleted taurine model on mitochondrial taurine and taurinomethyluridine content. It would also be of interest to evaluate the effect of severe taurine depletion on oxidative stress, which can arise from impaired mitochondrial protein synthesis secondary to taurine-deficient-mediated changes in tRNA^Leu^.

## Conclusions

In conclusion, our data show that β-alanine initiates a time-dependent decline in cellular taurine content, but does not cause a reduction in mitochondrial taurine levels or the 5-taurinomethyluridine content of mitochondrial tRNA^Leu^.

## Competing interests

The authors declare that they have no competing interests.

## Authors' contributions

CJJ and TI performed the experiments. MM, JA and SS were involved in drafting and revising the manuscript. All authors have read and approved the final manuscript.
